# Enrichment Characteristics of Hazardous Trace Elements in Feed Coal and Coal Ash in Huaibei Area under Leaching

**DOI:** 10.3390/toxics11040308

**Published:** 2023-03-26

**Authors:** Degao Wang, Jianwei Lu, Jian Wu, Bo Li, Ndhlovu Kataza Nyasha

**Affiliations:** 1 College of Geology and Construction Engineering, Anhui Technical College of Industry and Economy, Hefei 230051, China; 2 Key Laboratory of Intelligent Underground Exploration, College of Civil Engineering, Anhui Jianzhu University, Hefei 230601, China

**Keywords:** hazardous elements, enrichment characteristics, leaching behavior, Huaibei coalfield

## Abstract

This research focused on the hazardous elements in the main coal seam of Huaibei coalfield, China. Through collecting 20 feed coal samples from different coal seams of nine coal mines in the region, and combining with XRF, XRD, ICP-MS, and sequential chemical extraction, the mineral composition and the contents of major elements and HEs for feed coal were analyzed. Compared with previous research results, the enrichment characteristics of HEs in feed coal were revealed. The leaching behaviors of Se, Hg, and Pb in feed coal and coal ash under different leaching conditions were analyzed in depth by using a leaching device independently developed. Results showed that, compared with Chinese coals and World coals, the content of other elements, except Se, Sb, Hg, and Pb, in feed coal of Huaibei coalfield were at the “Normal” level, and no “Low” level elements were found; as the acidity of leaching solution decreased, the relative leaching rate of Se (*L_Se_*) was gradually increasing, while the *L_Hg_* and *L_Pb_* were not obvious; the *L_Se_* in feed coal and coal ash had a great relationship with the modes of occurrence of Se. The difference in the Hg content in the ion exchange state in feed coal may be an important reason for the difference in Hg leaching behavior. However, the content of Pb in feed coal had little influence on its leaching behavior. The modes of occurrence of Pb determined that the *L_Pb_* in feed coal and coal ash was not high. The *L_Se_* increased with the increase in acidity of leaching solution and leaching time. The leaching time was the main influencing factor of the *L_Hg_* and *L_Pb_*.

## 1. Introduction

According to the Yearbook of China’s National Bureau of Statistics, by the end of 2022, coal accounted for 56% of China’s primary energy consumption structure, and the environmental problems caused by coal burning are still of great importance and need to be urgently solved [1,2]. Although the content of hazardous elements (HEs) in coal is not high, it can accumulate in organisms or soil, posing a great threat to human health [3,4]. Affected by mining activities, the HEs in feed coal (FC) can be discharged to the surface through the transmission of groundwater, affecting the normal activities of surface organisms and human beings. Combustion products (coal ash (CA)) are the main industrial solid wastes of coal-fired power plants. Currently, the disposal method is mainly outcrop stacking, and the degree of resource utilization is not high [5]. In industrial activities, the CA can suspend in the air environment; be absorbed by the human body; cause air pollution; be affected by scouring, rain, and other natural influences; and can enter the surrounding soil and water to varying degrees, causing pollution of the surrounding water, soil, and crops [6,7].

There are many kinds of HEs in FC and CA, among which Se, Hg, Pb, and other elements are of great toxicity, volatility, and environmental sensitivity, and are important elements with great harm to the environment [8,9,10]. At present, more progress has been made in the study of leaching characteristics of HEs in FC and CA at home and abroad. In general, with the increase in leaching time, the concentration of HEs in leaching solution increases consistently [11]. The pH value and leaching time of leaching liquor are important factors affecting the leaching effect, and leaching rates of different pH values leaching liquor for different HEs are also different [12]. The difference of different environmental materials in leaching liquor will also have important influence on leaching results [13]. However, studying leaching characteristics of HEs in FC and CA is an effective method to evaluate their environmental hazards [14].

The leaching behaviors of Se, Hg, and Pb in FC and CA are summarized below [15,16].

### 1.1. Se

Although the content of Se element in FC is very low, it has been found that 10–50% of Se in a large number of CAs worldwide is extractable [17,18,19]. Previous studies have shown that Se in FC mainly exists in pyrite, followed by clay minerals and organic macerals. Chen et al. [20] observed, from leaching study of Se-containing high ash stone coal in the Enshi area, that the leaching solution pH value, liquid–solid ratio, and leaching time had little influence on the leaching effect of Se element, and the maximum leaching rate was not more than 2.5%. Through the study of leaching characteristics of Se in coal gangue and its burned slag and CA, it is concluded that the release of calcium in leaching liquor is an important factor determining the pH value of leaching liquor. Generally, the higher the pH value of leaching liquor, the higher the concentration of Se in CA and slag in leaching liquor, while the leaching amount of Se in coal gangue is inversely proportional to the pH value, i.e., leaching liquor controls the leaching of Se with acid corrosion of coal gangue. Therefore, when there is less precipitation in a certain area, the leaching rate of Se element increases, and the environmental risk also increases.

### 1.2. Hg

Hg is a highly volatile hazardous trace element. Coal burning is the main source of Hg emissions to the environment. Previous studies have shown that the occurrence of most Hg in FC is related to pyrite, followed by clay minerals and organic macerals. The level of Hg leaching reported in the literature indicates that the leaching of Hg from CA is not a major environmental problem. The leaching characteristics of Hg and As are relatively close, and are easy to leach out in the leaching process of CA. Compared with CA, the leaching effect of ash residue is better, and the leaching rate gradually reaches equilibrium within 4–5 h. According to the previous summary, it is concluded that temperature, types, and concentrations of complexes are important factors to control leaching characteristics of Hg and As elements.

### 1.3. Pb

As an element of sulfide in FC, Pb has obvious surface binding in CA. In acidic and alkaline CA samples, Pb is highly insoluble and almost immovable regardless of pH and leaching test, which may be related to the binding of the internal glass matrix of CA. Similar to other cations, acidic conditions slightly increased the leaching rate of Pb; however, the leachable concentration was still very low. According to [21], the fluidity of Pb seems to be controlled by phosphate minerals precipitation, and a phosphate mineral is highly insoluble in a wide pH range. The solubility of Pb is believed to decrease to a minimum around pH = 9, and then show a significant increase above pH = 11.5 due to the formation of a more soluble anionic water–oxygen complex [22]. Relevant scholars believe that no sample shows that the leaching level of Pb exceeds 0.06 mg/kg. Pb leaching generally does not pose a risk to the environment.

In summary, this study takes the HEs in the main mineable coal seam of Huaibei Coalfield as the research object, adopts the large data analysis method, and combines the test results of the content of HEs, revealing the enrichment characteristics of typical HEs in FC of Huaibei coalfield in China. In view of the high level of HEs (Se, Hg, and Pb) in FC and CA, the leaching characteristics and influencing factors of leaching solutions of these elements in three different pH values and four different times were analyzed by using a self-assembled leaching experimental device. Combined with the maximum allowable concentration limit of these elements in environmental standards, their environmental effects were evaluated.

The abbreviations that appear in the subsequent analysis of the article and their meanings are summarized in Table 1.

## 2. Sampling and Testing Methods

### 2.1. Sampling

**Figure 1:**
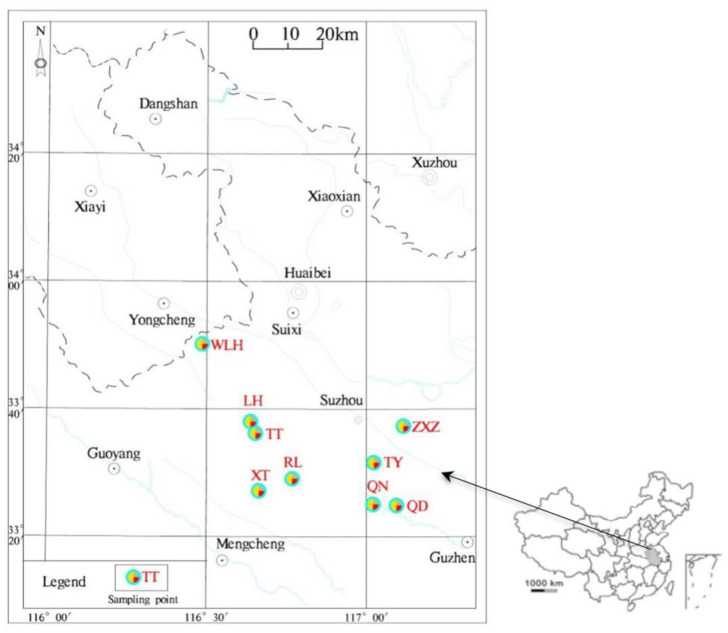
Location of FC sampling points in nine coal mines in Huaibei coalfield, China (Note: ZXZ: Zhuxianzhuang coal mine; TY: Taoyuan coal mine; QN: Qinan coal mine; QD: Qidong coal mine; RL: Renlou coal mine; XT: Xutuan coal mine; TT: Tongting coal mine; LH: Linhuan coal mine; WLH; Wolonghu coal mine).

The Huaibei coalfield is located in the coal-accumulation area of North China (Figure 1). The coal-bearing strata in the Huaibei coalfield are mainly the Permian Shanxi Formation (P_1_*s*), the Lower Shihezi Formation (P_2_*xs*), and the Shangshi Box Formation (P_2–3_*ss*). The total thickness of the coal-bearing strata is generally 750–850 m, and the coal content can reach more than 20 layers, in which the Shanxi Formation generally contains 1 coal group (10 coal groups), the Lower Shihezi group contains 5 coal groups (5, 6, 7, 8, and 9 coal groups), and the Upper Shihezi group mainly contains 2 coal groups (3 and 4 coal groups); other coal groups are usually not available.

In this study, 20 feed coal samples were collected from 9 mines in Huaibei coalfield (Figure 1). The sampling horizons are Lower Shihezi Formation and Shanxi Formation of Permian. The coal seams collected are the main coal seams currently being mined in each mine. The coal sample was collected at the underground working face. The method of sampling by grooving was used. The sample weight was more than 5 kg and is stored in a sealed bag.

### 2.2. Methods

#### 2.2.1. Proximate Analysis and Ultimate Analysis of FC

The proximate analysis volatile matter (V_daf_), moisture content (M_ad_), and ash yield (A_d_)) were performed according to ASTM D3172-1989. The ultimate analysis (carbon (C_daf_), hydrogen (H_daf_), and nitrogen (N_daf_)) were performed according to ASTM D5373-2008. The determination of total sulfur (S_t, d_) was carried out with reference to ASTM D2492-2002. The oxygen (O_daf_) was calculated by the difference method.

#### 2.2.2. Major Element Oxides of FC

The content of major element oxides (K_2_O, Na_2_O, SiO_2_, Al_2_O_3_, Fe_2_O_3_, CaO, MgO, TiO_2_, and MnO_2_) was determined by an X-ray fluorescence spectroscopy (Shimadzu, Kyoto, Japan, XRF-1800).

#### 2.2.3. Composition Analysis of Minerals in FC

The mineral compositions of FC were determined by X-ray diffractometer (PHILIPS X’Pert PRO) with Cu K-alpha radiation. The XRD pattern was recorded over a 2 θ interval of 10–70° with a step increment of 0.01°. The minerals are demarcated with reference to International Centre for Diffraction Data (ICDD) powder diffraction file.

#### 2.2.4. Sequential Chemical Extraction of FC

The chemical speciation of HEs (Se, Hg, and Pb) was determined based on the procedure described by [23]. The specific experimental process was presented in our earlier papers [24].

#### 2.2.5. Leaching Test of FC and CA

**Figure 2:**
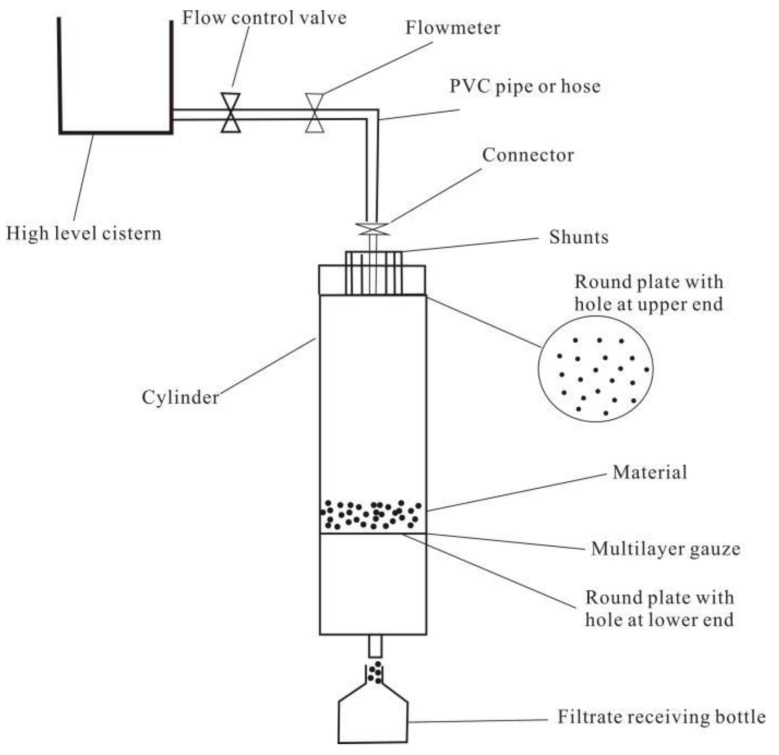
Schematic diagram of leaching simulation experiment device.

The leaching test equipment of FC and CA in this study was shown in Figure 2. The device was independently developed and designed by this research team.

The deionized water of pH = 2 was adjusted with dilute sulfuric acid on the acidity meter, and the deionized water of pH = 4 and pH = 7 was adjusted with dilute nitric acid and used as the three-leaching filtrate. The samples to be tested were pulverized to 100 mesh and uniformly stirred, and then 20.0 g of a diagonal sample was sampled into a leaching column by a quarter method. The various instruments used for leaching were soaked in 14% HNO_3_ for 24 h and rinsed with deionized water for use. The leaching column was fixed, and leaching was carried out with three kinds of leaching filtrates, respectively, so that the leaching filtrate with a height of about 5 cm was always maintained on the sample layer in the column, and the controlled flow rate was 3.0 mL/h. Into separate 25 mL, 25 mL, and 50 mL colorimetric tubes, and a 100 mL volumetric flask, the liquid was taken out at 5 h, 15 h, 30 h, and 60 h, rinsed with deionized water to the mark, shaken well, and then the liquid was prepared to be measured.

#### 2.2.6. Determination of HEs in FC and CA

Inductively coupled plasma mass spectrometry was employed to analyze the concentration of HEs in FC and CA. The experimental test was completed at the Physical and Chemical Science Experiment Center of the University of Science and Technology of China. The element concentration was tested using the X-Series 2 experimental equipment of Thermo Fisher Scientific, Waltham, MA, USA. The pre-treatment work was also carried out at the Physical and Chemical Science Experiment Center of the University of Science and Technology of China. The main process is as follows: firstly, approximately 2 g of FC sample with a fresh section was selected, ground, crushed, and sieved (200 mesh) to obtain a solid sample; the sample of CA was a temperature-programmed quartz tube. The furnace was prepared at a temperature of 900 °C for 30 min. The FC and CA were pretreated by heating and digestion methods. With a constant volume (25 mL), the experiment was completed at the Physical and Chemical Science Experiment Center of the University of Science and Technology of China. Two SARM20 coal standard samples were selected for the same experimental conditions and a blank test was added.

## 3. Results and Discussion

### 3.1. Basic Characteristics of Coal Quality in FC

Table 2 gives the basic data of coal quality of representative coal mines in Huaibei coalfield. According to Table 2, the moisture content of FC is generally between 0.68–2.2%. The A_d_ content is 15.69–21.38%. The V_daf_ content is between 12.64–35.44%. The ultimate analysis results of FC show that the percentage of C_daf_ is above 80%, followed by O_daf_ (6–9%), H_daf_ (5–7%), and N_daf_ (1–2%). The content of total S_t, d_ is usually low, generally 0.38–0.56%. The minerals in FC are mainly clay minerals, and the composition of CA is mainly SiO_2_ and Al_2_O_3_, followed by Fe_2_O_3_ and CaO. In summary, the basic characteristics of FC in the Huaibei coalfield are extremely low moisture content, low-medium ash content, medium-high volatile matter, and extremely low-low sulfur coal.

### 3.2. Enrichment Characteristics of Typical HEs in FC

Table 3 shows that the Cr content in the FC of the main coal seams (3-2, 6-1, 7-1, 7-2, 8, 9, and 10 coal seams) in the Huaibei coalfield is 23.31–99.50 μg/g, with an average of 39.97 μg/g; the Mn content is 4.29–47.50 μg/g, with an average of 17.18 μg/g; the Co content is 0.48–33.03 μg/g, with an average of 8.00 μg/g; the Ni content is 1.97–62.24 μg/g, with an average of 16.19 μg/g; the As content is 1.49–26.31 μg/g, with an average of 6.10 μg/g; the Se content is 1.99–20.40 μg/g, with an average of 10.37 μg/g; the Cd content is 0.11–0.74 μg/g, with an average of 0.31 μg/g; the Sb content is 0.49–35.39 μg/g, with an average of 9.31 μg/g; the Hg content is 0.04–0.14 μg/g, with an average of 0.1 μg/g; the Pb content is 4.95–134.83 μg/g, with an average of 35.30 μg/g/g. The content of Cr, Co, As, Se, Sb, and Pb is higher than that of Chinese coal and World coal (Table 4), Mn is lower than that of Chinese coal and World coal, and the content of other elements is between Chinese coal and World coal.

**Figure 3:**
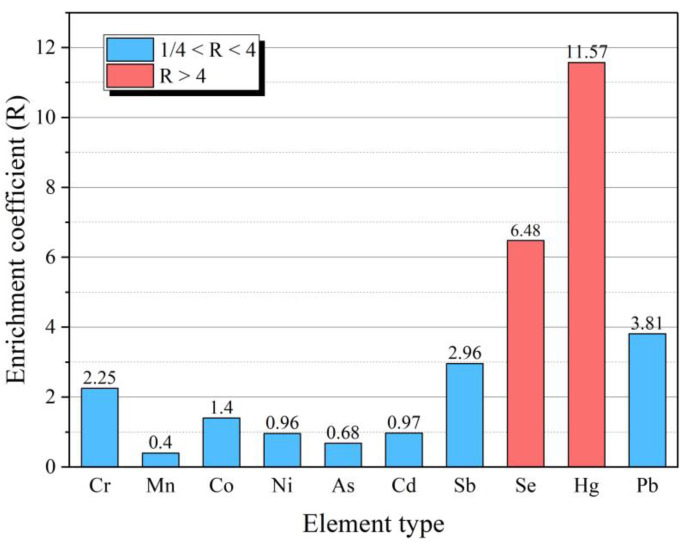
Enrichment coefficient of the studied HEs in FC.

In order to reflect the enrichment degree of HEs in FC in Huaibei coalfield, the ratio (R) of the arithmetic average value of the element content in FC to the average value of this element in World coal is taken as a mark to measure the element content level in FC in the study area (Table 4). R > 4 indicates a “High” content level, R < 1/4 indicates a “Low” content level, and the other is a “Normal” content level [31]. The enrichment coefficient (R) of HEs in FC is shown in Figure 3. Se and Hg are at “High” content level, and Pb is at “Normal” content level, but the R value is larger and closer to 4. Other elements are at “Normal” content level and none at “Low” content.

On the basis of the above analysis, we focused on the variation characteristics of Se, Hg, and Pb elements under different experimental conditions in the leaching test of FC and CA.

### 3.3. Leaching Characteristics of Se, Hg, and Pb in FC and CA

The leaching tests were carried out on typical RL-FC (7-2 coal), QD-FC (9 coal), WLH-FC (10 coal), and corresponding CA samples in the study area. Before the test, the contents of Se, Hg, and Pb in these samples are presented in Table 5.

#### 3.3.1. Calculation of Relative Leaching Rate

Table 6 shows the concentrations of HEs in leachate received under the conditions of three leaching media (pH = 2, 4, and 7) and four leaching time periods (*t* = 5 h, 15 h, 30 h, and 60 h). The constant flow rate of the leachate was 3.0 mL/h, so the volume of the leachate was the same for each time period. Based on the concentration of Se, Hg, and Pb elements in the leachate, the absolute dissolved amount of the above elements in different time periods and various leaching media can be calculated, and then the relative leaching rate *L_x_* is calculated:

*L_x_ = avt/AM* (1)

where *L_x_* is the leaching rate of element *x*; *a* is the concentration of *x* element in the leachate; *v* is the leaching rate; *t* is the leaching time; *A* is the concentration of *x* element in the analyzed sample (see Table 5); *M* is the total of the analyzed sample quality.

#### 3.3.2. Data Accuracy Analysis

In order to verify the accuracy of the data source in Table 6, the data in Table 6 were analyzed using a *t*-test. Take the Se leaching results of FC samples with pH = 2 and leaching time t = 30 h as an example. The results of all *T* values calculated for the *t*-test are shown in Table 7. The *T* values were obtained from the following sources:

(2) $$T_{i}=\frac{\left|L_{i}-\bar{L}\right|}{S}*\sqrt{n}$$

where, $L_{i}$ is the sample value of leaching rate, $\bar{L}$ is the average value of the sample value of the leaching rate, *n* is the number of determinations, the number of determinations in our study is 3, *n* = 3, and *S* is the standard deviation of the sample value of the leaching rate.

The 95% confidence level was chosen and the degree of freedom f = *n* − 1 = 2, and the *t*-test table was checked to know that the *T_t_* = 4.303. From Table 7, all the T values were less than 4.303, so the analysis method could be considered reliable. It should also be noted that the results in Table 6 are derived from the average of the three measurement results in Table 7. After verification by the *t*-test, the other data in Table 6 meet the requirements.

#### 3.3.3. Leaching Characteristics of Se Element in FC and CA

**Figure 4:**
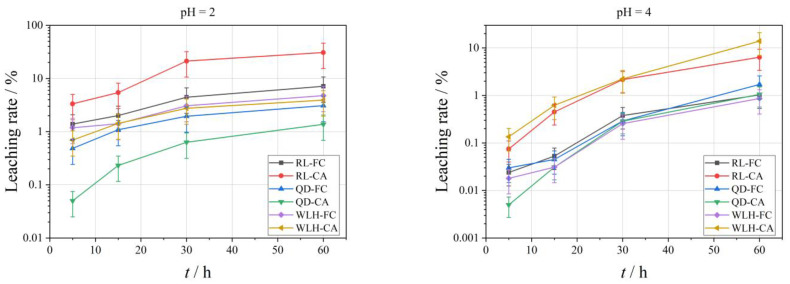

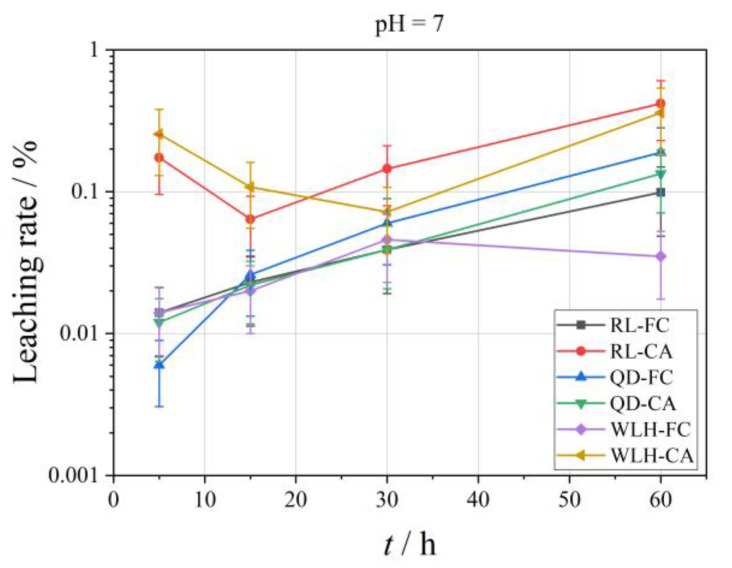
Leaching rate of Se in leaching solution under different leaching conditions.

The curve of the leaching rate of Se element under different pH conditions is shown in Figure 4. With the increase of leaching time, the leaching amount of different FC and CA samples showed an increasing trend. The specific performance is:

(1) With the decrease of acidity of leaching filtrate, the leaching effect of Se is characterized by increasing gradually, that is, the pH value rises from 2 to 7, and the maximum leaching rate (*L_se_*_, max_) of Se is reduced from 30.73% to 0.42%.
(2) Compared with FC, the relative leaching rate (*L_se_*) of Se in CA is relatively high, i.e., the *L_se_*_, max_ in FC is 0.19–7.12% under different pH conditions; the *L_se_*_, max_ in CA is 0.13–30.73%.
(3) The *L_se_*_, max_ in different types of samples is also different, and the overall performance is RL-CA > RL-FC > WLH-CA > WLH-FC > QD-FC > QD-CA.
(4) With the increase of leaching time, the overall trend of *L_se_* in FC and CA are increasing. Generally, the *L_se_* is relatively high before 30 h, and then increases slowly from 30 h to 60 h with a trend of continuous growth.

#### 3.3.4. Leaching Characteristics of Hg Element in FC and CA

**Figure 5:**
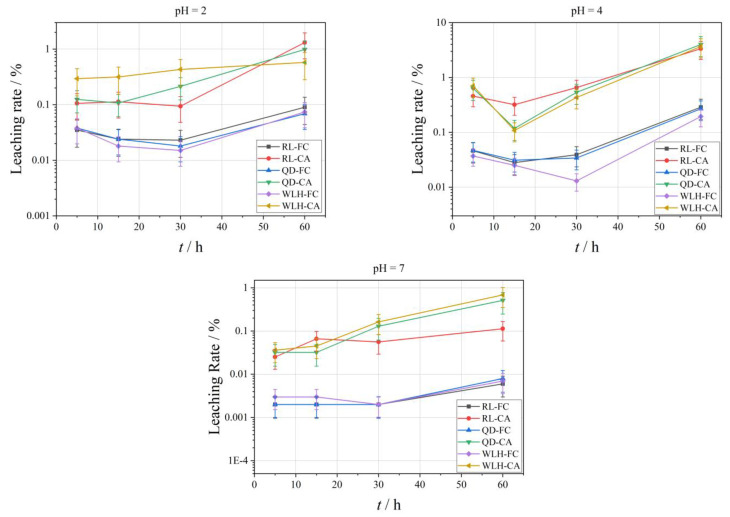
Leaching rate of Hg in the leaching solution under different leaching conditions.

The curve of the relative leaching rate (*L_Hg_*) of Hg element under different pH conditions is shown in Figure 5. With the increase in leaching time, the leaching amount of different FC and CA samples shows an increasing trend. Specific performance is as follows:

(1) With the decrease in acidity of the leaching filtrate, the leaching effect of the Hg element is not obvious, that is, the maximum leaching rate (LHg, max) of Hg is 0.99% when pH = 2; the LHg, max is 3.99% when pH = 4; the LHg, max is 0.68% when pH = 7.
(2) Relative to FC, the leaching rate of Hg in CA is relatively big (same order of Se), i.e., the LHg, max in FC is 0.006–0.29% under different pH conditions; the LHg, max in CA is 0.11–3.99%.
(3) With the increase in leaching time, the LHg in FC shows a decreasing trend in the first 30 h when pH = 2, and an increasing trend from 30 to 60 h. When pH = 4, the LHg in FC shows a decreasing trend in the first 15 h and an increasing trend from 15 to 60 h; when pH = 7, there is an overall increasing trend from 5 to 60 h.

#### 3.3.5. Leaching Characteristics of Pb Element in FC and CA

**Figure 6:**
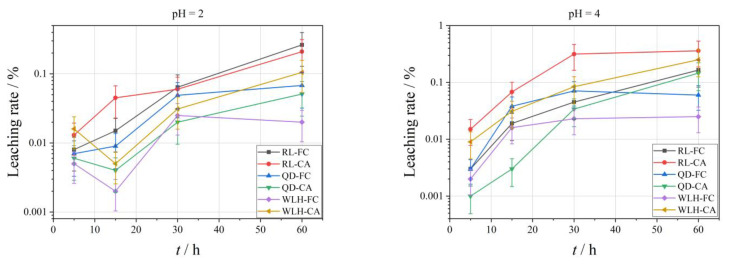

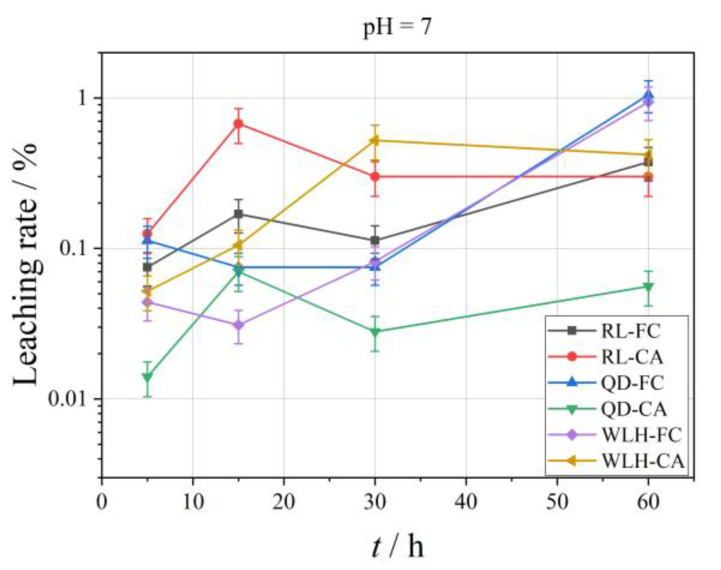
Leaching rate of Pb in the leaching solution under different leaching conditions.

Figure 6 shows the change curve of the relative leaching rate (*L_Pb_*) of Pb element under different pH conditions. As the leaching time increases, the leaching amount of different FC and CA samples shows an increasing trend. Specific performance is as follows:

(1) The *L_Pb_* in different pH leaching filtrates is not high, and the maximum leaching rate (*L_Pb_*_, max_) of Pb was less than 2%. When pH = 2, the *L_Pb_*_, max_ is 0.26%; when pH = 4, the *L_Pb_*_, max_ is 0.36%; when pH = 7, the *L_Pb_*_, max_ is 1.05%.
(2) Compared with FC, the *L_Pb_* in CA is relatively high (one order of magnitude higher), and under different pH conditions, the *L_Pb_*_, max_ in FC is 0.03–1.05%; the *L_Pb_*_, max_ in CA is 0.05–0.68%. The specific properties are different at different pH values and at different time periods at the same pH, as can be seen from the figure.
(3) At pH = 2, *L_Pb_* shows an increasing trend for RL-FC, RL-CA and QD-FC, a decreasing trend for QD-CA, WLH-FC, and WLH-CA in the time range of 5–15 h, and an increasing trend for all of them in the time range of 15–60 h. *L_Pb_* shows an increasing trend in the time range of 5–60 h at pH = 4. Under the condition of pH = 7, *L_Pb_* shows a trend of increasing from 5 to 15 h, decreasing from 15 to 30 h, and increasing from 30 to 60 h for RL-FC, RL-CA, and QD-CA; however, *L_Pb_* shows an overall increasing trend for QD-FC, WLH-FC, and WLH-CA.

### 3.4. Analysis of Factors Affecting Leaching Behavior of Se, Hg, and Pb in FC and CA

There are many factors affecting the leaching and precipitation of HEs from FC and CA. The internal factors mainly include the content and occurrence state of HEs in FC and CA, the mineral composition of FC, etc. The external factors mainly include the pH value of leaching filtrate and the leaching time. This study attempts to reveal the main controlling factors and internal relations affecting the leaching behavior of FC and CA by establishing the relationship between the *L*_max_ of HEs in FC and CA and the above factors (Table 8).

#### 3.4.1. The Relationship between the Content of Se, Hg, and Pb and Their Modes of Occurrence

##### Se

**Figure 7:**
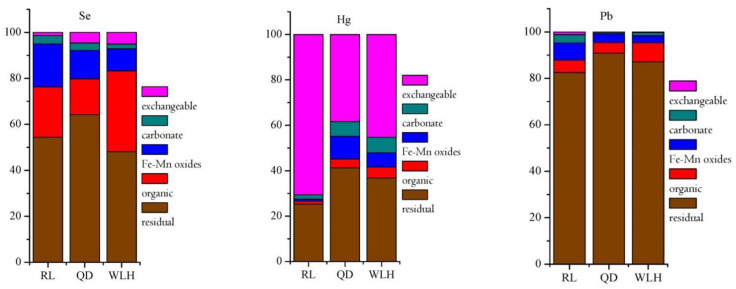
The modes of occurrence of Se, Hg, and Pb in FC.

According to Pearson correlation coefficient (*r*) (see Table 8) of HEs content and *L*_max_ in FC and CA, the content of Se element in FC and CA is closely related to the *L*_*Se*, max_ (*r* = 0.938 and *r* = 0.518, respectively). More generally, the higher the content of Se in FC and CA, the better the leaching effect of Se, which may be related to the occurrence state of Se. Se can either adsorb selenate [32] or combine with iron oxide [33]. However, this weak binding makes Se easy to release, and it may react with calcium to form stable compounds (sulfate-like properties) no matter how Se is present in the coal matrix [34]. Figure 7 shows that the modes of occurrence of Se element in FC is mainly residue state (48.08–64.21%), organic state (15.56–35.14%), and Fe-Mn oxidation state (9.60–18.74%), while the carbonate binding state is less (2.10–3.68%). The anomaly of the above results may be related to the low metamorphic grade and low sulfide content of the coal samples in the study area [35]. It can be seen that the leaching effect of Se element in FC and CA has a great relationship with the occurrence state of Se. Generally, the smaller the Se content in the carbonate-bound state, the more favorable the leaching precipitation of Se element.

##### Hg

The content of Hg in FC and CA is also closely related to the *L*_*Hg*, max_ (*r* = 0.807 and *r* = 0.665, respectively). However, according to Pflughoeft-Hassett [36] and Sanchez et al. [37], the relationship between Hg content and leaching rate has been vague. The leaching of Hg appears to be controlled by adsorption from the aquatic phase [37], which may be related to the difference in the occurrence state of Hg. Figure 7 shows that the modes of occurrence of Hg element in different FCs is obviously different. The occurrence state of Hg is mainly the ion exchange state (38.40–70.58%) and residue state (25.31–41.24%), followed by the iron and manganese oxidation state (0.84–10.06%), carbonate binding state, and organic state, which indicate that Hg is primarily present in silicate minerals and is water-soluble state. Therefore, it can be seen that the higher the Hg content in FC and CA, the greater the leaching rate of Hg and the difference in the content of Hg in the ion exchange state of FC may be an important cause of the difference in leaching behavior of Hg.

##### Pb

The relationship between the *L*_*Pb*, max_ and the content of Pb in FC is not obvious (*r* = −0.451), but it has a positive correlation with the content of Pb in CA (*r* = 0.647). As an element of sulfide in coal, Pb has significant surface bonding in CA [38]. In acidic and alkaline fly ash samples, Pb is highly insoluble and almost immobile regardless of pH and leaching tests [18,39,40,41,42] may be associated with glass matrix bonding within CA [43]. Figure 7 shows that the modes of occurrence of Pb element in FC is mainly residue state (82.51–90.82%), followed by organic combined state (4.60–8.13%) and iron-manganese oxidation state (3.13–7.45%), which indicates that the Pb element mainly exists in the silicate minerals and has a small content in the sulfide. Therefore, the occurrence state of Pb determines that the *L_Pb_* in FC and CA is not high, while the content of Pb element in coal has little influence on the leaching and precipitation of this element.

#### 3.4.2. The Relationship between Mineral Composition of FC and Leaching of Se, Hg, and Pb

**Figure 8:**
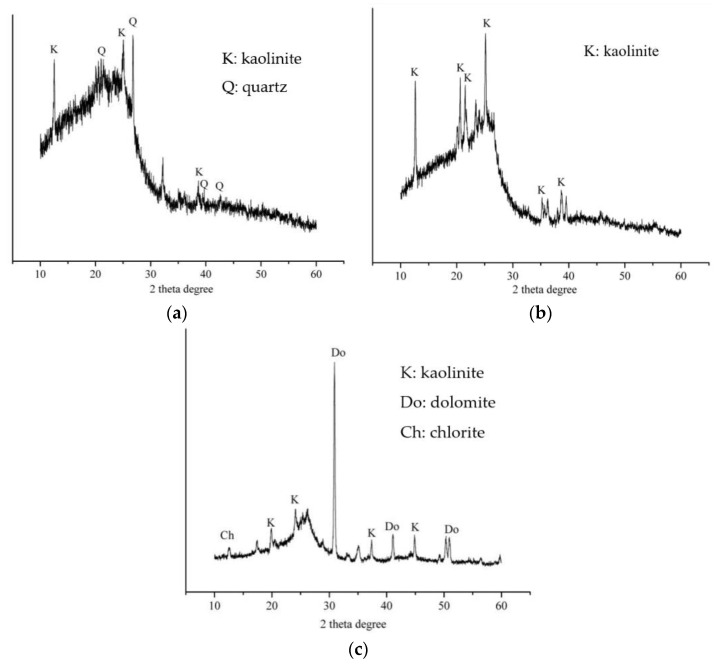
The XRD diffraction patterns of FC in main coal seam of three typical coal mines. In the figure, (**a**) is 7-2 coal, RL (Renlou coal mine); (**b**) is 9 coal, QD (Qidong coal mine); (**c**) is 10 coal, WLH (Wolonghu coal mine).

Figure 8 indicates that the mineral composition of 7-2 coal (RL) in the Lower Shihezi formation of the Huaibei coalfield is mainly kaolinite and quartz, and the mineral composition of 9 coal (QD) is mainly kaolinite. The diffraction peaks of other minerals are not obvious. The mineral composition of Shanxi formation 10 coal (WLH) is mainly kaolinite and quartz. The XRD pattern of this coal has obvious diffraction peaks of dolomite minerals, which may be related to the igneous intrusion into the coal seam.

**Figure 9:**
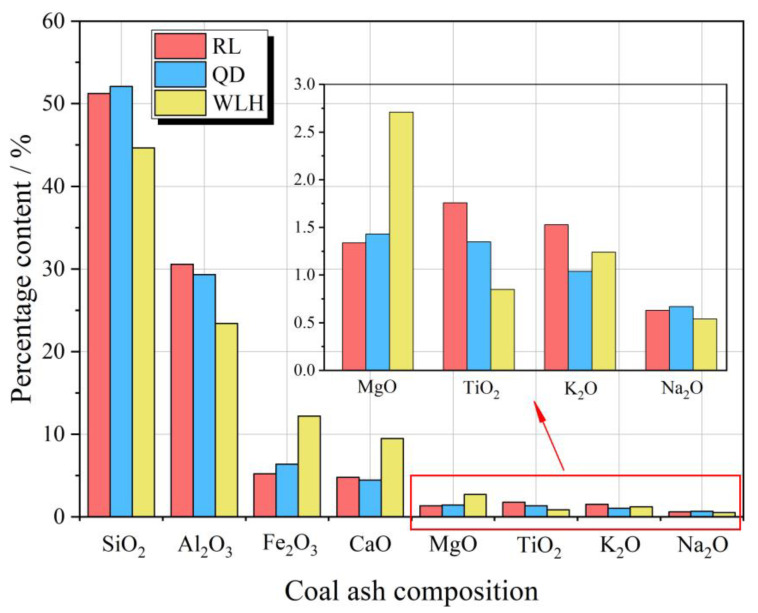
Main element composition of FC ashed in the main coal seam of three typical coal mines. In the figure, RL: Renlou coal mine; QD: Qidong coal mine; WLH: Wolonghu coal mine.

In addition, Figure 9 shows that the CA composition of each main coal seam is dominated by SiO_2_ and Al_2_O_3_, with SiO_2_ content ranging from 44.64% to 52.08%, Al_2_O_3_ content ranging from 23.42% to 30.57%, and a small amount of Fe_2_O_3_ (5.22–12.20%) and CaO (4.45–9.47%). According to Pearson correlation coefficient ® (Table 9) between S_t, d_, Fe_2_O_3_, and CaO in the FC and the maximum leaching rate, the leaching rate of Se and Hg in FC and AC has a very high correlation with S_t, d_ and shows a negative correlation, while Pb is not obvious, suggesting that the sulfide content in coal inhibits the leaching rate of Se and Hg, while the Pb in FC is poor in solubility. According to Nathan et al. [39], Ward et al. [18], Moreno et al. [42], Dubikova et al. [21], and Izquierdo et al. [38], the leaching level of Pb in FC is difficult to exceed 0.06 mg/kg. Therefore, the change of total sulfur and ash composition in FC has little influence on the leaching rate of Pb. In addition, the correlation between the content of Fe_2_O_3_ and CaO in CA and the maximum leaching rate of Se, Hg, and Pb elements is not obvious. According to the analysis, this may be related to the lower content of Fe_2_O_3_ and CaO in FC in the study area and the fact that the above elements (Se, Hg and Pb) are mainly associated with clay minerals [44,45].

#### 3.4.3. The Relationship between pH Value, Leaching Time, and Leaching of Se, Hg, and Pb

**Figure 10:**
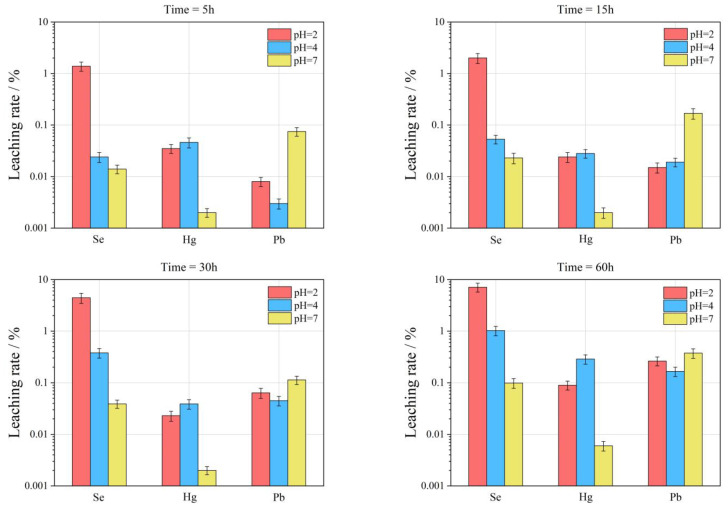
Variation of leaching rates of Se, Hg, and Pb in the RL-FC under different pH values and leaching time conditions.

In general, the leaching rate of the same element increases with the increase of leaching time as the acidity of leaching solution increases. As can be seen from Figure 10, under the same leaching time, the more acidic the leaching solution is, the more the Se element leaching rate is, while the Hg and Pb elements are affected by their own chemical properties; this rule is not obvious. However, with the increase of leaching time, the leaching rates of Se, Hg, and Pb all increased gradually. For the Se element, the leaching effect is best when pH = 2, the maximum leaching rate in 60 h can reach 7.12%, and leaching equilibrium is not reached. The Hg element has the best leaching effect when pH = 4, and the maximum leaching rate in 60 h is only 0.29%, which also indirectly shows that the difference of Hg content in ion-exchange state in FC is an important reason for the change of leaching rate. The leaching rate of Pb under different pH conditions has little difference, indicating that the change in the pH value of leaching liquor has little influence on the leaching rate of Pb. In a word, the leaching rate of the Se element is obviously affected by pH and leaching time. The leaching time is the main factor affecting the leaching rate of Hg and Pb.

**Figure 11:**
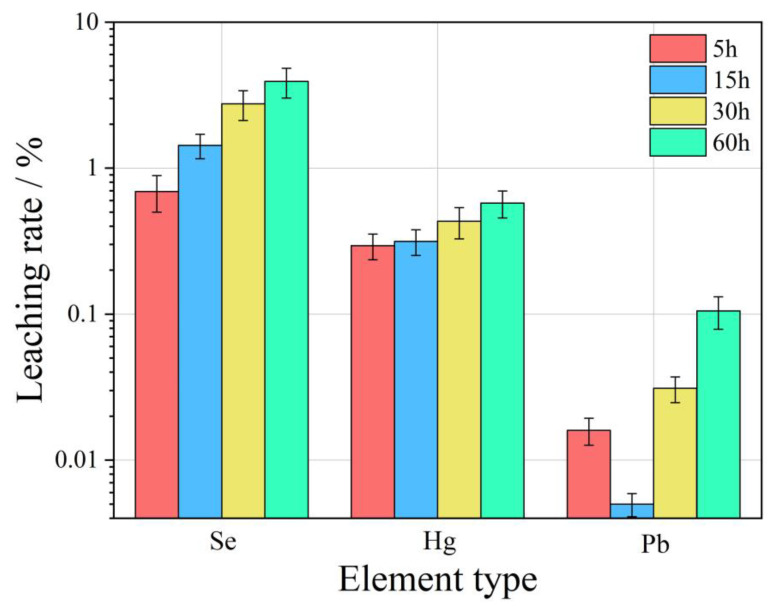
Variation of leaching rates of Se, Hg, and Pb in the WLH-CA under different leaching time conditions.

Globally, the changes in Se, Hg, and Pb leaching rates in WLH-CA under different leaching time conditions are presented in Figure 11. It can be seen from the figure, in general, thst the leaching rate of the same element increases with the increase in leaching time. The leaching rates of both Se and Hg elements increase with the increase in leaching time, and the leaching rate of Pb tends to decrease during 5–15 h and increase within 15–60 h.

Overall, this study provides important information for coal mining and utilization in the Huaibei area. The results show the key role of acidity and leaching time on the leaching of hazardous trace elements from coal. By reducing the hazardous elements discharged from coal transported from groundwater to the surface during mining, the accumulation of hazardous substances in soil and aquatic systems will likely be reduced, thereby minimizing the impact on organisms and normal human activities. The research results reveal the enrichment characteristics and leaching behavior of hazardous elements Se, Hg, and Pb in the main coal seams of the Huaibei area.

## 4. Conclusions

In this paper, the hazardous elements in coal seams of Huaibei area were taken as the research object, and 20 feed coal samples collected were analyzed by combining research methods such as XRF, XRD, ICP-MS, and sequential chemical extraction. Through leaching experiments on hazardous elements Se, Hg, and Pb, the following conclusions were obtained:

(1) The content of Cr, Co, Se, Sb, Hg, and Pb in FC from Huaibei coalfield was higher than that of Chinese coals and World coals. Except for Se and Hg, the content of other elements was at “Normal” levels, but the content of Pb was also larger. The above results may be related to the unique stratigraphy of the Huaibei coalfield, where the coal-bearing strata are the upper and lower Shi Box Formation and Shanxi Formation of the Paleozoic Permian, and the upper part of the coal-bearing strata is covered by loose layers of the Cenozoic.
(2) The general trend indicated that the leaching rate of the same element increased with the increase in leaching time. With the decrease in acidity of the leaching solution, the leaching rate of the Se element was gradually increasing, while the leaching rates of Hg and Pb element were not obvious. This situation may be related to the state of occurrence of selenium, which can adsorb selenate as well as combine with iron oxide. In addition, the leaching of mercury seems to be controlled by the adsorption of the aquatic phase. In acidic and alkaline fly ash samples, lead is highly insoluble and hardly moves, which may be the reason for the insignificant leaching rate of mercury and lead.
(3) The leaching rate of the Se element had a great relationship with its modes of occurrence. The difference in the Hg content in the ion-exchange state may be the main reason for the difference in the Hg leaching rate. The content of Pb had little effect on its leaching rate, while its modes of occurrence (residue state) determined its leaching rate. This may be due to the low metamorphic grade and low sulfide content of the coal samples in the study area. It can also be seen that the higher the Hg content in FC and CA, the greater the leaching rate of Hg and the difference in the Hg content in the ion exchange state may be an important reason for the difference in the leaching behavior of Hg; the occurrence state of Pb determines that the *L_Pb_* in FC and CA is not high, and the content of Pb element in coal has little effect on the leaching and precipitation of this element.
(4) The *L_Se_* was controlled by the pH value and leaching time of the leaching solution at the same time, specifically, the lower the pH value and the longer the leaching time, the more the *L_Se_*; the *L_Hg_* and *L_Pb_* mainly depended on leaching time. Therefore, the low pH of the coalfield environment and the increase in the leaching rate of Se, Hg, and Pb contained in the coal seam over time will lead to an increase in heavy metal content in the surrounding environmental soil and damage the ecological environment.

## Figures and Tables

**Table 1:** Abbreviations Summary Table.

Abbreviation	Description
CA	coal ash
FC	feed coal
HEs	hazardous elements
*L_Se_*	the relative leaching rate of Se
*L_Pb_*	the relative leaching rate of Pb
*L_Hg_*	the relative leaching rate of Hg
V_daf_	volatile matter
M_ad_	moisture content
A_d_	ash yield
C_daf_	carbon
H_daf_	hydrogen
N_daf_	nitrogen
S_t, d_	total sulfur
O_daf_	oxygen
RL	Renlou coal mine
QD	Qidong coal mine
WLH	Wolonghu coal mine

**Table 2:** Basic information of FC in the main coal seam of three typical coal mines in the Huaibei coalfield.

**Sample**	**Coal** **Seam**	**Proximate Analysis** **(wt. %)**			**Ultimate Analysis** **(wt. %)**				**Total Sulfur** **Content** **(wt. %)**	**Major Elements Analysis** **(wt. %)**							
		M_ad_	A_d_	V_daf_	C_daf_	H_daf_	N_daf_	O_daf_	S_t, d_	SiO_2_	Al_2_O_3_	Fe_2_O_3_	CaO	MgO	TiO_2_	K_2_O	Na_2_O
RL	7-2	2.06	19.38	35.44	85.53	5.65	1.35	6.98	0.38	51.23	30.57	5.22	4.78	1.34	1.76	1.53	0.63
QD	9	0.68	21.38	32.59	83.98	5.34	1.38	8.22	0.56	52.08	29.32	6.38	4.45	1.43	1.35	1.04	0.67
WLH	10	2.2	15.69	12.64	82.35	6.92	1.24	8.83	0.51	44.64	23.42	12.20	9.47	2.71	0.85	1.24	0.54

Note: RL (Renlou coal mine); QD (Qidong coal mine); WLH (Wolonghu coal mine); ad: air drying free; daf: dry-ash-free; d: dry-free.

**Table 3:** The HEs content test results of 20 FC samples collected from nine coal mines in the Huaibei coalfield.

Sample	Coal Mine	Coal Seam	Cr (μg/g)	Mn (μg/g)	Co (μg/g)	Ni (μg/g)	As (μg/g)	Se (μg/g)	Cd (μg/g)	Sb (μg/g)	Hg (μg/g)	Pb (μg/g)
QD-1	Qidong	3-2	25.14	67.80	15.40	18.00	nd	nd	0.13	0.92	0.48	15.28
QD-2	Qidong	6	43.41	22.33	9.92	22.68	26.31	5.54	0.55	3.30	0.11	16.11
QD-3	Qidong	7	99.50	47.50	33.03	62.24	11.26	6.86	0.74	5.28	0.14	37.82
QD-4	Qidong	9	26.02	9.60	7.58	23.30	2.77	1.99	0.11	0.81	0.04	4.95
ZXZ-1	Zhuxianzhuang	8	28.64	12.57	2.45	14.63	6.67	3.20	0.12	0.49	0.06	7.10
ZXZ-2	Zhuxianzhuang	10	23.31	8.82	9.50	16.77	4.45	2.31	0.21	1.44	0.05	11.46
RL-1	Renlou	7-2	36.36	20.33	16.26	36.08	11.66	5.98	0.25	3.41	0.12	10.05
RL-2	Renlou	8-2	27.05	25.51	9.83	21.79	6.02	5.50	0.35	2.73	0.11	10.42
WLH-1	Wolonghu	8	35.43	5.82	6.43	19.56	13.06	6.93	0.16	0.84	0.14	14.29
LH-1	Linhuan	7	nd	11.33	6.23	12.23	2.16	14.63	nd	10.99	nd	19.75
LH-2	Linhuan	8	nd	30.23	4.07	7.23	2.38	16.29	nd	6.86	nd	121.26
LH-3	Linhuan	9	nd	4.29	0.48	7.57	3.64	12.94	nd	7.36	nd	62.61
TT-1	Tongting	9	nd	nd	4.21	13.35	1.49	11.33	nd	8.45	nd	79.10
TY-1	Taoyuan	8	nd	29.03	1.62	4.25	4.55	20.40	nd	35.39	nd	26.00
TY-2	Taoyuan	10	nd	4.73	nd	1.97	2.28	17.48	nd	20.78	nd	28.02
QN-1	Qinan	6-1	nd	8.56	7.75	13.24	3.69	12.10	nd	8.85	nd	134.83
QN-2	Qinan	7-1	nd	nd	4.24	6.78	3.77	9.69	nd	9.35	nd	9.14
QN-3	Qinan	7-2	nd	nd	nd	2.70	2.11	11.12	nd	9.88	nd	38.90
XT-1	Xutuan	7-1	nd	17.44	6.18	10.71	3.60	15.39	nd	15.96	nd	23.17
XT-2	Xutuan	7-2	nd	16.78	6.32	10.51	4.06	17.27	nd	24.72	nd	15.73

Note: In the table, “nd” means below detection limit.

**Table 4:** Comparison of HEs in FC from the Huaibei coalfield with Chinese coals and World coals.

Elements	Measured Data of Huaibei Coalfield (μg/g)	Collecting Data of Huaibei Coalfield ^a^ (μg/g)	Chinese Coals ^b^ (μg/g)	World Coals ^c^ (μg/g)
Cr	$\frac{23.31-99.50}{38.32}$	$\frac{15-201}{50}$	$\frac{0.1-942.7}{15.4}$	$\frac{0.5-60}{17}$
Mn	$\frac{4.29-67.80}{20.16}$	nd	271.2 *	50 *
Co	$\frac{0.48-33.03}{8.42}$	nd	$\frac{0.1-59.3}{7.08}$	$\frac{0.5-30}{6}$
Ni	$\frac{1.97-62.24}{16.28}$	nd	$\frac{0.5-186}{13.7}$	$\frac{0.5-50}{17}$
As	$\frac{1.49-26.31}{6.10}$	$\frac{0.4-2.5}{1.4}$	$\frac{0-478.0}{3.79}$	$\frac{0.5-80}{9}$
Se	$\frac{1.99-20.40}{10.37}$	nd	$\frac{0.02-82.2}{2.7}$	$\frac{0.2-10}{1.6}$
Cd	$\frac{0.11-0.74}{0.29}$	nd	0.46 *	0.30 *
Sb	$\frac{0.49-35.39}{8.89}$	nd	2.56 *	3.0 *
Hg	$\frac{0.04-0.48}{0.14}$	nd	1.37 *	0.012 *
Pb	$\frac{4.95-134.83}{34.30}$	nd	$\frac{0.2-790}{15.1}$	$\frac{2-80}{9}$

Note: the range of HEs is above the horizontal line and the average value is below; ^a^: data from Liu et al. [25]; ^b^: data from Ren et al. [26] and Dai et al. [27]; ^c^: data from Swaine [28], Ketris and Yudovich [29]; *: data from Zhao [30].

**Table 5:** Contents of HEs in FC and CA of a representative three coal mines before the leaching experiment.

Sample Number		Se (μg/g)	Hg (μg/g)	Pb (μg/g)
FC	RL-FC	5.98	0.12	10.05
	QD-FC	1.99	0.04	4.95
	WLH-FC	2.31	0.15	11.46
CA	RL-CA	1.32	0.10	2.98
	QD-CA	1.25	0.06	1.97
	WLH-CA	0.98	0.05	3.36

**Table 6:** Relative leaching rates of three HEs under different leaching conditions.

Leachate		pH = 2				pH = 4				pH = 7			
Leaching Time		Time (h)				Time (h)				Time (h)			
		5	15	30	60	5	15	30	60	5	15	30	60
Se	RL-FC	1.386	2.007	4.438	7.115	0.024	0.053	0.378	1.025	0.014	0.023	0.039	0.099
	RL-CA	3.335	5.432	21.263	30.729	0.075	0.450	2.154	6.364	0.174	0.064	0.145	0.418
	QD-FC	0.485	1.085	1.946	3.089	0.030	0.045	0.291	1.708	0.006	0.026	0.060	0.189
	QD-CA	0.050	0.231	0.631	1.374	0.005	0.031	0.288	1.057	0.012	0.022	0.039	0.134
	WLH-FC	1.186	1.406	3.069	4.763	0.018	0.031	0.255	0.865	0.014	0.020	0.046	0.035
	WLH-CA	0.693	1.431	2.754	3.924	0.135	0.621	2.232	13.968	0.255	0.108	0.072	0.360
Hg	RL-FC	0.035	0.024	0.023	0.090	0.046	0.028	0.039	0.287	0.002	0.002	0.002	0.006
	RL-CA	0.106	0.113	0.094	1.313	0.456	0.319	0.656	3.338	0.025	0.066	0.056	0.113
	QD-FC	0.038	0.024	0.018	0.069	0.047	0.031	0.034	0.268	0.002	0.002	0.002	0.008
	QD-CA	0.125	0.107	0.214	0.986	0.636	0.118	0.536	3.986	0.032	0.032	0.129	0.514
	WLH-FC	0.038	0.018	0.015	0.074	0.037	0.025	0.013	0.194	0.003	0.003	0.002	0.007
	WLH-CA	0.294	0.315	0.432	0.576	0.702	0.108	0.432	3.672	0.036	0.045	0.162	0.684
Pb	RL-FC	0.008	0.015	0.064	0.263	0.003	0.019	0.045	0.165	0.075	0.169	0.113	0.375
	RL-CA	0.013	0.045	0.060	0.210	0.015	0.068	0.315	0.360	0.125	0.675	0.300	0.300
	QD-FC	0.007	0.009	0.049	0.068	0.003	0.038	0.071	0.060	0.113	0.075	0.075	1.050
	QD-CA	0.006	0.004	0.020	0.051	0.001	0.003	0.034	0.146	0.014	0.070	0.028	0.056
	WLH-FC	0.005	0.002	0.025	0.020	0.002	0.016	0.023	0.025	0.044	0.031	0.082	0.941
	WLH-CA	0.016	0.005	0.031	0.105	0.009	0.031	0.084	0.251	0.052	0.105	0.523	0.419

**Table 7:** Summary of T_i_ values when using the *t*-test.

Sample Number	RL-FC		QD-FC		WLH-FC	
	Data/(μg/g)	*T_i_* (*i* = 1, 2, 3)	Data/(μg/g)	*T_i_* (*i*=1, 2, 3)	Data/(μg/g)	*T_i_* (*i* = 1, 2, 3)
Results	4.123	2.081	1.656	2.189	3.159	0.748
	4.426	0.079	1.965	0.143	3.267	1.646
	4.765	2.160	2.217	2.046	2.781	2.394
Average of the data	4.438		1.946		3.069	
SD of the data	0.262		0.229		0.208	

Note: the “SD” means the standard deviation, *T_i_* (*i* = 1, 2, 3) is the *T* value corresponding to the data of the three determinations.

**Table 8:** Pearson correlation coefficient of HEs content and maximum leaching rate in FC and CA.

Correlation Coefficients (*r*)	Sample Type	
	FC	CA
Correlation Coefficients with Maximum Leaching Rate (*L*_max_)		
Se	0.938	0.518
Hg	0.807	0.665
Pb	−0.451	0.647

Note: Significant at 0.05 levels (2-tailed).

**Table 9:** Pearson correlation coefficient of S_t, d_, Fe_2_O_3_, and CaO in FC and maximum leaching rate.

Correlation Coefficients (*r*)		Total Sulfur	Oxide	
		S_t, d_	Fe_2_O_3_	CaO
Correlation Coefficients with Maximum Leaching Rate (*L*_max_)				
Se	FC	−0.988	−0.250	−0.038
	CA	−0.995	−0.483	−0.286
Hg	FC	−0.981	−0.568	−0.380
	CA	−0.928	−0.026	0.187
Pb	FC	0.396	0.504	0.309
	CA	0.191	−0.229	−0.017

Note: Significant at 0.05 levels (2-tailed).

## Data Availability

Not applicable.

## References

[1] Chen Z., Qian F., Chen D. (2015). Evaluation of the use of coal-based synthetic natural gas for haze prevention in China. J. Environ. Sci. China.

[2] Li W., Ma Z., Huang Q., Jiang X. (2018). Distribution and leaching characteristics of heavy metals in a hazardous waste incinerator. Fuel.

[3] Dai S., Ren D., Chou C., Finkelman R., Seredin V., Zhou Y. (2012). Geochemistry of trace elements in Chinese coals: A review of abundances, genetic types, impacts on human health, and industrial utilization. Int. J. Coal Geol..

[4] De Vallejuelo S., Gredilla A., da Boit K., Teixeira E., Sampaio C.H., Madariaga J., Silva L. (2017). Nanominerals and potentially hazardous elements from coal cleaning rejects of abandoned mines: Environmental impact and risk assessment. Chemosphere.

[5] Ahmaruzzaman M. (2010). A review on the utilization of fly ash. Prog. Energy Combust..

[6] Dreher K., Jaskot R., Lehmann J., Richards J., McGee Andrew J., Costa G. (2010). Soluble transition metals mediate residual oil fly ash induced acute lunginjury. J. Toxicol. Environ. Health A.

[7] Kostova I., Vassileva C., Dai S., Hower J. (2016). Mineralogy, geochemistry and mercury content characterization of fly ashs from the Maritza 3 and Varna thermoelectric power plants, Bulgaria. Fuel.

[8] Liu J., Zheng B., Aposhian H., Zhou Y., Chen M., Zhang A., Waalkes M. (2002). Chronic arsenic poisoning from burning high-arsenic-containing coal in Guizhou, China. J. Peripher. Nerv. Syst..

[9] Wang T., Wang J., Burken J., Ban H., Ladwig K. (2007). The leaching characteristics of selenium from coal fly ashes. J. Environ. Qual..

[10] Yin L., Zhou Y., Xu Q., Zhu Z., Du W., An Z. (2013). Mercury emission from coal-fired power plants in China. Proc. Chin. Soc. Electr. Eng..

[11] Neupane G., Donahoe R. (2013). Leachability of elements in alkaline and acidic coal fly ash samples during batch and column leaching tests. Fuel.

[12] Dutta B., Khanra S., Mallick D. (2009). Leaching of elements from coal fly ash: Assessment of its potential for use filling abandoned coal mines. Fuel.

[13] Khanra S., Mallick D., Dutta S., Chaudhuri S. (1998). Studies on the phase mineralogy and leaching characteristics of coal fly ash. Water Air Soil Pollut..

[14] Zheng L., Ding S., Liu C. (2016). Leaching characteristics of environmentally sensitive trace elements in different types of coal gangue. J. Cent. South Univ. (Nat. Sci. Ed.).

[15] Hassett D., Pfughoeft-hassett D., Heenink L. (2005). Leaching of CCBs: Observations from over 25 years of research. Fuel.

[16] Christenson H., Pope J., Trumm D., Newman N., Blanco I., Kerr G., Young M., Uster B. (2019). Manganese and trace element removal from new zealand coal mine drainage using limestone leaching beds. N. Zeal. J Geol. Geophys..

[17] Nugteren H., Janssen-Jurkovícová M., Scarlett B. (2001). Improvement of environmental quality of coal fly ash by applying forced leaching. Fuel.

[18] Ward C., French D., Riley K., Stephenson L., Farrell O., Li Z. (2011). Element leachability from a coal stockpile and associated coastal sand deposits. Fuel Process. Technol..

[19] Iwashita A., Sakaguchi Y., Nakajima T., Takanashi H., Ohki A., Kambara S. (2005). Leaching characteristics of boron and selenium for various coal fly ashes. Fuel.

[20] Chen Z., Wu J., Qiao Y., Xu M. (2013). Combustion characteristics of Enshi stone soal and leaching characteristics of heavy metal of Se element. J. Huazhong Univ. Sci. Technol. (Nat. Sci.Ed.).

[21] Ward C.R., French D., Jankowski J., Dubikova M., Li Z., Riley K.W. (2009). Element mobility from fresh and long-stored acidic fly ashes associated with an Australian power station. Int. J. Coal Geol..

[22] Jones D., Swaine D.J., Goodarzi F. (1995). The Leaching of Major and Trace Elements from Coal Ash. Environmental Aspects of Trace Elements in Coal.

[23] Tessier A., Campbell P., Blsson M. (1979). Sequential extraction procedure for the speciation particulate trace metals. Anal. Chem..

[24] Fu B., Liu G., Mian M., Sun M., Wu D. (2019). Characteristics and speciation of heavy metals in fly ash and FGD gypsum from Chinese coal-fired power plants. Fuel.

[25] Liu G., Zheng L., Duzgoren-Aydin N., Gao L., Liu J., Peng Z. (2007). Health effects of arsenic, fluorine, and selenium from indoor burning of Chinese coal. Rev. Environ. Contam. Toxicol..

[26] Ren D., Zhao F., Dai S., Zhang J. (2006). Trace Element Geochemistry of Coal.

[27] Dai S., Wang X., Zhou Y., Hower J., Li D., Chen W., Zhu X., Zou J. (2011). Chemical and mineralogical compositions of silicic, mafic, and alkali tonsteins in the late Permian coals from the Songzao Coalfield, Chongqing, Southwest China. Chem. Geol..

[28] Swaine D. (1994). Trace elements in coal and their dispersal during combustion. Fuel Process. Technol..

[29] Ketris M., Yudovich Y. (2009). Estimations of Clarkes for Carbonaceous biolithes: World averages for trace element contents in black shales and coals. Int. J. Coal Geol..

[30] Zhao F. (1997). Experimental Study on Distribution and Occurrence Mechanism of Hazardous Trace Elements in Coal and Leaching of Coal Products.

[31] Ren D., Xu D., Zhao F. (2004). A preliminary study on the enrichment mechanism and occurrence of hazardous trace elements in the Tertiary lignite from the Shenbei coalfield, China. Int. J. Coal Geol..

[32] Yudovich Y., Ketris M. (2006). Selenium in coal: A review. Int. J. Coal Geol..

[33] Riley K., French D., Lambropoulos N., Farrell O., Wood R., Huggins F. (2007). Origin and occurrence of selenium in some Australian coals. Int. J. Coal Geol..

[34] Freyer D., Voigt W. (2003). Crystallization and phase stability of CaSO4 and CaSO4-based salts. Monatshefte Chem..

[35] Finkelman R., Palmer C., Wang P. (2018). Quantification of the modes of occurrence of 42 elements in coal. Int. J. Coal Geol..

[36] Pflughoeft-Hassett D. (2004). Leaching Characteristics of Fly Ash–Activated Carbon from Mercury Control Technologies.

[37] (2006). Characterization of Mercuryenriched Coal Combustion Residues from Electric Utilities Using Enhanced Sorbents for Mercury Control.

[38] Izquierdo M., Koukouzas N., Touliou S., Panopoulos K., Querol X., Itskos G. (2011). Geochemical controls on leaching of lignite-fired combustion by-products from Greece. Appl. Geochem..

[39] Nathan Y., Dvorachek M., Pelly I., Mimran U. (1999). Characterization of coal fly ash from Israel. Fuel.

[40] Praharaj T., Powell M., Hart B., Tripathy S. (2002). Leachability of elements from sub-bituminous coal fly ash from India. Environ. Int..

[41] Kim A., Kazonich G., Dahlberg M. (2003). Relative solubility of cations in class F fly ash. Rev. Environ. Sci. Biol..

[42] Moreno N., Querol X., Andrés J., Stanton K., Towler M., Nugteren H., Janssen-Jurkovicová M., Jones R. (2005). Physico-chemical characteristics of European pulverized coal combustion fly ashes. Fuel.

[43] Warren C., Dudas M. (1988). Leaching behaviour of selected trace elements in chemically weathered alkaline fly ash. Sci. Total Environ..

[44] Tewalt S., Bragg L., Finkelman R. Mercury in U.S. Coal—Abundance, Distribution, and Modes of Occurrence. U.S. Geological Survey Fact Sheet. 2001, FS–095–01. http://pubs.usgs.gov/fs/fs095-01/index.html.

[45] Hower J., Robertson J. (2003). Clausthalite in coal. Int. J. Coal Geol..

